# The local regulation of folliculogenesis by members of the transforming growth factor superfamily and its relevance for advanced breeding programmes

**DOI:** 10.21451/1984-3143-AR2018-0055

**Published:** 2018-08-16

**Authors:** Jennifer L. Juengel, Peter R. Smith, Laurel D. Quirke, Michelle C. French, Sara J. Edwards

**Affiliations:** 1 Reproduction, Animal Science, AgResearch Ltd., Invermay Agricultural Centre, Mosgiel New Zealand.

**Keywords:** granulosa cells, oocyte, theca cells

## Abstract

Regulation of the growth and maturation of the ovarian follicle is critical for normal reproductive function. Alterations in this growth can lead to pathological conditions, such as cystic follicles, reduced oocyte quality, or an abnormal endocrine environment leading to poor fertility. Alterations in follicular growth also influence the number of follicles ovulating and thus can change litter size. Both endocrine factors, such as follicle stimulating hormone and luteinizing hormone, as well as local factors, are known to regulate follicular growth and development. This review will focus on the role of local factors in regulation of ovarian follicular growth in ruminants, with a focus on members of the transforming growth factor superfamily. The potential role of these factors in regulating proliferation, apoptosis, steroidogenesis and responsiveness to gonadotrophins will be considered.

## Introduction

At formation, the ovarian follicle consists of the female germ cell, the oocyte, surrounded by a single layer of support cells, the granulosa cells. Once follicular growth has been initiated, follicular development can be characterised through proliferation of the granulosa and recruitment and proliferation of additional support cells (theca cells). The oocyte continues to grow and mature, and the granulosa and theca cells differentiate to be able to communicate with the hypothalamus, pituitary and reproductive tract and respond to endocrine factors controlling ovulation, final maturation of the oocyte, and luteinisation for subsequent progesterone production for maintenance of early pregnancy. Another important aspect of this development is factors controlling atresia of the ovarian follicle, as the majority of ovarian follicles follow this pathway rather than the pathway to ovulation. It is known that follicles at any developmental stage can become atretic, from a primordial follicle right through to a follicle that had developed to a preovulatory size but failed to ovulate and regressed ( Matsuda *et al*., 2012 ). However, it is also clear that while fewer follicles are classified as atretic during preantral follicular development, many follicles become atretic around or shortly after the time of formation of the antrum ( Driancourt *et al*., 1985 ).

A large proportion of follicular development can occur without pituitary hormones. For instance in sheep, follicles will grow to approximately 3 mm in diameter after the pituitary has been removed ( McNatty *et al*., 1990 ). Once a follicle has reached 3 mm in diameter, the remaining growth to obtain a preovulatory size (approximately 5-6 mm) can occur in a matter a days. Considering that normal development of a follicle to ovulation has been estimated to take six months, clearly the majority of follicular growth is independent of gonadotrophins. Similar results are observed in cattle, with LH only required once a follicle reaches a diameter of 8-9 mm and FSH likely required for follicles over 4 mm ( Mihm and Bleach, 2003 ). Ovulation occurs in bovine follicles around 15 mm in diameter although this varies with age and breed ( Pohler *et al*., 2012 ). It should be noted however, that while the earlier stages of follicular development are not dependent on the gonadotrophins, these small growing follicles are responsive to both FSH and LH. Receptors (R) for FSH are expressed in the granulosa cells starting during preantral development and LHR are present on theca cells starting in late preantral/early antral follicles ( Tisdall *et al*., 1995 ; Bao and Garverick, 1998 ; Logan *et al*., 2002 ; Saraiva *et al*., 2011 ; Barros *et al*., 2013 ).

**Figure 1:**
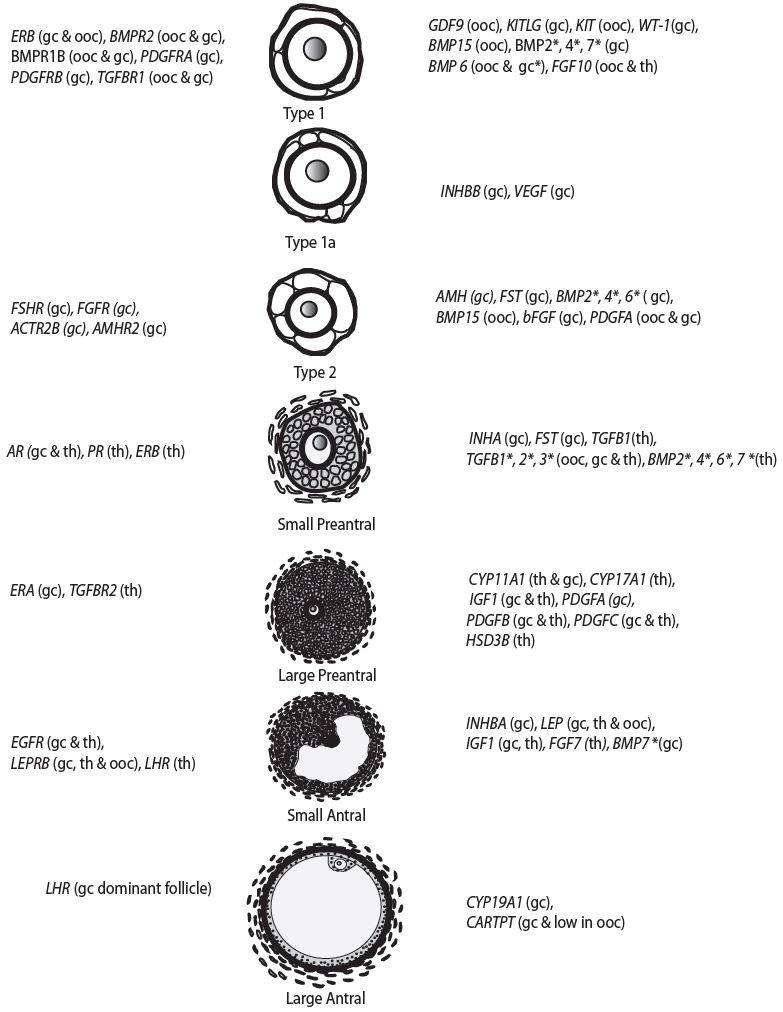
The stages of follicular development and initiation of expression of selected proteins in the healthy follicle. Newly formed follicles can consist of either an oocyte surrounded by a single layer of granulosa cells with a flattened morphology (termed a type 1 or primordial follicle) or an oocyte surrounded by a single layer of granulosa cells with a mixture of flattened and cuboidal granulosa cells (termed a type 1a or transitional follicle; Juengel and Smith, 2014 ). The granulosa cells and the oocyte are isolated within a basement membrane and thus separated from the ovarian vasculature. Soon after follicles are formed, they begin to grow, forming a type 2 or primary follicle, which contain a single layer of cuboidal granulosa cells ( Lundy *et al*., 1999 ). Type 3 or small preantral contains 2 to < 4 complete layers of granulosa cells whereas a type 4 or large preantral follicle contains at least 4 complete layers of granulosa cells but no antral cavity is yet present. The theca can first be observed in some type 2 follicles and becomes very prominent in type 4 follicles ( Lundy *et al*., 1999 ). These theca cells are located outside the basement membrane of the ovarian follicle and contain a rich blood supply. Patterns of expression are based on mRNA or protein localisation in either sheep, cattle or goat ovaries. Once expression is initiated, it is also observed in subsequent stages of development although concentrations may vary. ooc= oocyte, gc = granulosa cell, th = theca. An asterisk (*) indicates known differences in expression patterns between species with expression in sheep tending to be more restricted than that observed in cattle and goats. Please see text for more details. Information on expression of proteins was gathered from multiple references ( Bezard *et al*., 1987 ; Wandji *et al*., 1992 ; Braw-Tal, 1994 ; Tisdall *et al*., 1994 1995 1997 ; Leeuwenberg *et al*., 1995 ; Xu *et al*., 1995 ; Logan *et al*., 2002 2003 ; Juengel *et al*., 2004a 2006a b ; Kobayashi *et al*., 2004 ; Buratini *et al*., 2007 ; Feary *et al*., 2007 ; Brito *et al*., 2012 ; Lima *et al*., 2012 ; Smith, 2012 ; Batista *et al*., 2013 ; Hao *et al*., 2014 ; Diaz *et al*., 2016 ).

Multiple locally produced factors have been identified as controlling the development of the ovarian follicle. These locally produced factors ( Fig. 1 ) include, but are not limited to, members of the transforming growth factor beta (TGFB) super family, insulin-like growth factors (IGF), fibroblast growth factors (FGF), platelet derived growth factors (PDGF) and KIT ligand (KITL). Additionally, the TGFB superfamily consists of multiple sub-families, including the growth and differentiation factors (GDF), bone morphogenetic proteins (BMP), activins and inhibin, TGFB and anti-mullerian hormone (AMH). In this review we will focus on members of the GDF, BMP and TGFB sub-groups of the TGFB superfamily. Readers are directed to additional reviews for information on other factors controlling ovarian follicular development ( Young and McNeilly, 2010 ; Buratini and Price, 2011 ; Scaramuzzi *et al*., 2011 ; Knight *et al*., 2012 ; Campbell *et al*., 2014 ; Knight and Glister, 2014 ; Monniaux *et al*., 2014 ; Price, 2016 ; Shimizu, 2016 ; Silva *et al*., 2016 ; Pankhurst, 2017 ; Estienne and Price, 2018 )

## The effects of TGFB superfamily members on granulosa cell proliferation and health ( Fig. 2 )

**Figure 2:**
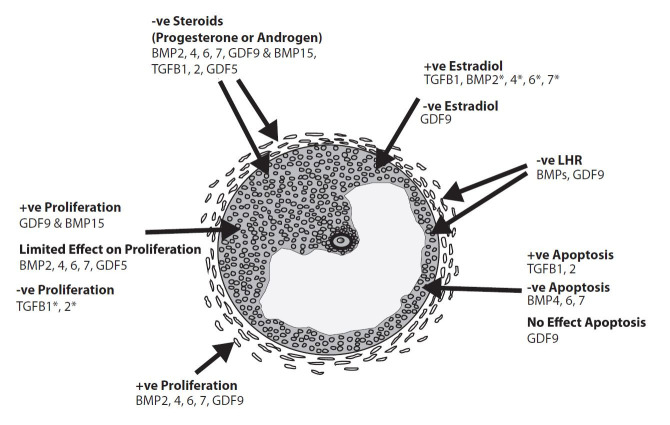
Summary of the effects of BMPs, GDFs and TGFBs on granulosa and theca cell proliferation and function. +ve = positive effect, -ve = negative effect. An asterisk (*) indicates differences have been observed in responses between species (sheep vs. cattle) or studies within species. Please see text for more details.

Two oocyte-secreted growth factors from the TGFB superfamily, GDF9 and BMP15, are known to be essential for normal granulosa cell proliferation. In ewes homozygous for inactivating mutations in either growth factor, normal follicular development does not occur, and follicles are blocked in the early preantral stages of development ( Davis *et al*., 1992 ; Braw-Tal *et al*., 1993 ; Galloway *et al*., 2000 ; Nicol *et al*., 2009 ). Immunisation against GDF9 or BMP15 can also affect follicular development in both sheep and cattle, resulting in reduced numbers of antral follicles which were smaller in size ( Juengel *et al*., 2002 , 2009 , 2011 ). Treatment of granulosa cells from small antral follicles, primarily prior to gonadotrophin dependence (i.e. from follicles 1-2 mm in sheep and 1-4.5 mm in cattle) with ovine (o) BMP15 or the combination of oBMP15 and oGDF9 induced thymidine incorporation indicative of proliferation ( McNatty *et al*., 2005 ).

Other BMPs and GDFs are also produced by the ovarian follicles, although their role in regulating local follicular development is less clear and may vary between species. The oocyte also produces BMP6 in sheep ( Juengel *et al*., 2006b ) and cattle ( Hussein *et al*., 2005 ) but BMP6 did not induce cell proliferation in sheep ( Juengel *et al*., 2006b ) and had a small effect on bovine mural granulosa cells ( Glister *et al*., 2004 ) and no effect on cumulus cells ( Gilchrist *et al*., 2006 ). In cattle, granulosa and theca cells from small antral follicles (1-4 mm in diameter) produce BMP2, BMP4, BMP6 and BMP7 ( Glister *et al*., 2010 ) whereas in sheep, as determined by *in situ* hybridisation, only granulosa cells of atretic follicles express BMP2. The other BMPs were not expressed by the granulosa or theca cells, although BMP4 was produced in ovarian stroma cells surrounding some follicles. In sheep, none of these growth factors stimulated proliferation of the granulosa cells ( Souza *et al*., 2002 ; Campbell *et al*., 2006 ; Juengel *et al*., 2006b ) although one study showed a small stimulation of proliferation when examining BMP4 ( Fabre *et al*., 2003 ). In cattle neither BMP2 nor 4 affected cell proliferation ( Glister *et al*., 2004 ; Selvaraju *et al*., 2013 ), with BMP6 and 7 promoting a small increase in viable granulosa cell numbers ( Glister *et al*., 2004 ). The effects of GDF5 on granulosa cell proliferation has been examined with no effect observed ( Fabre *et al*., 2003 ).

In sheep, TGFB1 and TGFB2, but not TGFB3, are produced by ovarian follicles, in the theca cells of type 3 and larger follicles ( Juengel *et al*., 2004a ). In cattle, TGFB1, 2 and 3 were all detected in oocytes, granulosa and theca cells, with TGFB3 being the most strongly expressed ( Nilsson *et al*., 2003 ). TGFB1 has also been detected in oocytes of goat preantral follicles ( Rodrigues *et al*., 2014 ). TGFB1 and 2 both reduced numbers of granulosa cells after culture in sheep ( Juengel *et al*., 2004a ), although no effect of TGFB1 on proliferation was observed (TGFB2 was not tested). In cattle, the effects of TGFB are inconsistent, with TGFB2 having a mild stimulatory effect on proliferation ( Gilchrist *et al*., 2003 ), and TGFB (type unspecified) having no effect on unstimulated, or an inhibitory effect on epidermal growth factor (EGF)-stimulated, proliferation ( Skinner *et al*., 1987 ).

Members of the BMP family, including BMP4, 6, 7 and 15 have been linked to reduced cumulus/mural granulosa cell apoptosis in cattle ( Hussein *et al*., 2005 ; Kayamori *et al*., 2009 ). In bovine granulosa cells, BMP4 suppression of apoptosis was linked to the PI3K/PDK-1/Akt pathway whereas BMP7 suppression of apoptosis was linked to the PI3K/PDK-1/PKC pathway ( Shimizu *et al*., 2012 ). In bovine preantral follicles, culture with BMP15 alone stimulated follicle growth without reducing viability whereas culture with the combination of FSH and BMP15 resulted in reduced numbers of viable follicles, potentially linked to over stimulation of proliferation of granulosa cells ( Passos *et al*., 2013 ). In contrast, GDF9 had no effect on apoptosis in cattle cumulus cells ( Hussein *et al*., 2005 ). Further evidence for the role of members of the BMP superfamily in regulating apoptosis is provided by the observation that overexpression of the regulatory micro(mi)RNA-375 decreases expression of BMPR2 and increases apoptosis of bovine cumulus cells ( Chen *et al*., 2017 ). This receptor is key for the actions of many BMPs including the synergistic actions of BMP15 and GDF9 ( Edwards *et al*., 2008 ). Additionally, reduction of SMAD2 expression, through either chi-miRNA-4110 mimics or SMAD2 interference, reduced SMAD2 mRNA and protein in caprine granulosa cells and increased apoptosis ( An *et al*., 2017 ). The SMAD2/3 pathway is important for the synergistic actions of ovine BMP15 and GDF9 ( Reader *et al*., 2011 ). In contrast to the suppressive effects of some BMP family members on apoptosis, members of the TGFB subfamily may actually induce apoptosis in ruminants. As indicated before, treatment with TGFB1 or 2 reduced DNA content after culture in sheep granulosa cells ( Juengel *et al*., 2004a ), and TGFB1 increases apoptosis in cattle granulosa cells ( Zheng *et al*., 2009 ).

## The effects of TGFB superfamily members on theca cell proliferation and health ( Fig. 2 )

Less is known regarding regulation of theca cell function. As indicated previously, the theca cells express many members of the TGFB superfamily and their receptors in sheep, cattle and goats ( Glister *et al*., 2004 , 2010 ; Juengel *et al*., 2004a , 2006b ; Feary *et al*., 2007 ; Costa *et al*., 2012 ; Lima *et al*., 2012 ; Rodrigues *et al*., 2014 ). BMP2, 4 and 6 stimulated theca cell proliferation in sheep, with the effects of BMP7 not tested ( Campbell *et al*., 2006 ). In cattle, BMP4, 6 and 7 stimulated theca cell proliferation in the presence or absence of LH, while BMP2 was not tested ( Glister *et al*., 2005 ). GDF9 stimulates proliferation of theca cell isolated from small, but not larger, antral follicles ( Spicer *et al*., 2008 ).

## TGFB superfamily regulation of granulosa and theca cell function ( Fig. 2 )

One of the mechanisms that many of the locally produced growth factors use to alter ovarian follicular development during antral development is through regulating the follicle’s ability to communicate with the hypothalamus and pituitary. In particular, regulation of steroids and inhibin A produced by the ovarian follicle, and receptors for the gonadotrophins, appear to be key mechanisms by which local factors affect the development of the ovarian follicle and, ultimately, the number of follicles available for ovulation. To this end, many of the local growth factors alter steroid synthesis and gonadotrophin responsiveness ( Fig. 2 ).

### Regulation of steroidogenesis

In ewes with reduced BMP15 function (i.e. those heterozygous for an inactivating mutation in *BMP15* such as Inverdale) or altered BMP signalling (i.e. those heterozygous or homozygous for a mutation in *BMPR1B,* that is the Booroola mutation), a key feature is that oestrogen active follicles are observed at smaller sizes. Additionally, BMP4, 6 and 7 suppress basal and LH-stimulated androgen production from bovine theca cells ( Glister *et al*., 2005 ). Suppression of expression of genes involved in steroidogenesis such as *CYP17A1* (strongly suppressed), *STAR*, *CYP11A1* and *HSD3B1* mRNA were observed. This is consistent with the BMP family members suppressing steroidogenesis through downregulation of proteins important for steroid production. GDF9 also reduced both progesterone and androstenedione production from bovine theca cells ( Spicer *et al*., 2008 ). Decreased expression of mRNA encoding *LHCGR* and *CYP11A1* was observed following GDF9 treatment, but no effects on *STAR* or *CYP17A1* mRNA were observed ( Spicer *et al*., 2008 ). In ovine theca cells, the BMPs (2, 4 and 6 tested) inhibited LH-stimulated androstenedione production ( Campbell *et al*., 2006 ). Consistent with this observation, ewes that were heterozygous for both the Inverdale and Booroola mutations (I+B+), tended to have increased expression of *CYP17A1* , which is expressed exclusively in the theca in ovine ovarian follicles ( Logan *et al*., 2002 ), in small (1-3 mm in diameter) follicles ( Juengel *et al*., 2017 ).

The effects of BMPs on steroid production in granulosa cells is complex. In both sheep and cattle granulosa cells, BMP2, 4, 6, and 7, as well as the combination of BMP15 & GDF9 strongly suppressed progesterone production when IGF was included in the media ( Fabre *et al*., 2003 ; Glister *et al*., 2004 ; McNatty *et al*., 2005 ; Juengel *et al*., 2006b ). A weaker suppression of basal progesterone production was also observed when examined in cattle ( Glister *et al*., 2004 ) and sheep ( Pierre *et al*., 2004 ). In sheep, BMP4 (only ligand tested) decreased expression of mRNA and protein for *STAR* and *CYP11A1*, likely through inhibiting the actions of *SF1* ( Pierre *et al*., 2004 ). TGFB1 and 2, GDF5 and GDF9 also reduced progesterone production from ovine or bovine granulosa cells ( Fabre *et al*., 2003 ; Juengel *et al*., 2004a ; Spicer *et al*., 2006 ). In bovine granulosa cells, the TGFB1 induced reduction of progesterone production was associated with downregulation of *STAR*, *CYP11A1* , and *HSD3B1* mRNAs ( Zheng *et al*., 2008 ). However, various BMPs have also been shown to either increase FSH and IGF1 stimulated oestradiol production ( Glister *et al*., 2004 ; Campbell *et al*., 2006 ; Selvaraju *et al*., 2013 ), or have no effect, or suppress oestradiol production, dependent on the dose of both the BMP and the IGF ( Campbell *et al*., 2006 ). TGFB1 also increased basal oestradiol synthesis and the expression of *CYP19A1* and *HSD17B1* ( Zheng *et al*., 2009 ). GDF9 suppressed oestradiol production from bovine granulosa cells collected from both small and large antral follicles ( Spicer *et al*., 2006 ). Thus overall, BMPs, GDFs and TGFB appear to inhibit progesterone production from granulosa cells, likely through down regulation of proteins important for steroid synthesis. The effects of the superfamily members on oestradiol production is less consistent, with potential for differences observed being related to species, which family member is being examined and dose of growth factor used.

The effects of mutations in BMP15 or BMP1B in ewes, and increased expression of *SMAD6 *, an inhibitory SMAD for the BMP pathway (i.e. the Trio cattle which have a genetic based increase in ovulation rate), on steroid secretion from follicles and cells has also been examined. Granulosa cells from homozygous Booroola animals secreted increased concentrations of oestradiol and theca cells secreted increased concentrations of androstenedione ( Campbell *et al*., 2006 ), indicative of enhanced oestrogen production. It is important to note that the follicles in homozygous Booroola animals mature at a smaller size than wild-type and thus follicles of similar sizes likely have differing maturation status. However, this difference was at least partially accounted for as smaller diameter follicles were collected from the Booroola animals ( Campbell *et al*., 2006 ). Expression of *CYP19A1* mRNA was increased in small follicles from ewes heterozygous for the Booroola mutation (as well as one copy of the Inverdale mutation). However, whether the Booroola mutation increases or decreases the sensitivity of the receptor to BMPs is unclear, with some studies observing an increased sensitivity ( Campbell *et al*., 2006 ; Young *et al*., 2008 ), and others a decreased sensitivity ( Fabre *et al*., 2003 ). Potentially further complicating this model is the finding that *BMP15* mRNA is decreased in homozygous Booroola ewes ( Crawford *et al*., 2011 ). In the Trio animals, with overexpression of *SMAD6* in cattle follicles ( Kamalludin *et al*., 2018 ), a similar phenotype to that observed in ewes carrying the Booroola or Inverdale mutation, with ovulation of multiple smaller follicles, is seen ( Garcia-Guerra *et al*., 2018a , b ). In the Trio animals, with follicles of similar size, increased oestradiol concentrations in follicular fluid are also observed ( Garcia-Guerra *et al*., 2018b ).

In heterozygous Inverdale ewes, overall secretion of oestradiol from all the preovulatory follicles is similar to that observed in wild-type ewes. However, the follicles in the Inverdale ewe are smaller with fewer granulosa cells in each follicle, and with more follicles contributing to the overall pool of granulosa cells secreting oestradiol (and inhibin) to influence the hypothalamus and pituitary ( Shackell *et al*., 1993 ; Juengel *et al*., 2013a ). No differences in concentrations of oestradiol in follicular fluid were noted when comparisons were based on follicular diameter. The secretory capacity of each granulosa cell for oestradiol or inhibin appeared similar between heterozygous Inverdale and wild-type ewes ( Shackell *et al*., 1993 ).

Further evidence of the role of the BMP family in normal follicular function is provided by the observation that in cattle, alterations in expression of BMP4, BMP6 and BMPR1B occur during the development of ovarian cysts ( Diaz *et al*., 2016 ). Development of ovarian cysts is known to be related to alterations in apoptosis and steroid production in the ovarian follicle ( Ortega *et al*., 2015 ).

### Effects on responsiveness to FSH and LH

A key feature of granulosa cells from ewes carrying Inverdale and/or Booroola mutations, thus likely lower BMP15 activity, is an earlier onset of responsiveness to LH. This has been observed when examining the mRNA encoding *LHCGR* or the ability of the cells to bind and respond to LH, as assessed by cAMP production ( McNatty *et al*., 2009 , 2017 ; Crawford *et al*., 2011 ; Juengel *et al*., 2017 ). Similarly, in cows carrying a copy of the Trio mutation, which have increased expression of mRNA encoding the inhibitory *SMAD6* ( Kamalludin *et al*., 2018 ), thus postulated reduced BMP signalling, follicles have an earlier responsiveness to LH with increased expression of the LHR in the granulosa cells ( Garcia-Guerra *et al*., 2018a ). Treatment of bovine theca cells with BMPs also reduces LH stimulated androstenedione production, indicative of reduced responsiveness to LH ( Glister *et al*., 2005 ). In contrast, theca cells isolated from small antral follicles collected from homozygous carriers of the Booroola mutation produced more androstenedione when stimulated with low doses of LH ( Campbell *et al*., 2006 ) compared to non-carriers. However, basal secretion was also increased and thus responsiveness was not greater. Additionally, BMPs inhibited LH stimulated androstenedione production in ovine theca cells ( Campbell *et al*., 2006 ). Thus, collectively, it appears BMPs inhibit LH responsiveness of follicular cells.

The role of BMPs in regulating FSHR expression is less clear. FSH responsiveness, as measured by mRNA encoding *FSHR* or the ability of the cells to bind and respond to FSH in general, is not different between control ewes and ewes carrying the Inverdale and/or Booroola mutations ( McNatty *et al*., 2009 , 2017 ; Crawford *et al*., 2011 ; Juengel *et al*., 2017 ). Similarly, cows carrying the Trio mutation do not have differential expression of *FSHR* mRNA compared to non-carrier controls ( Garcia-Guerra *et al*., 2018a ). However, *FSHR* mRNA was increased in preantral bovine follicles cultured with hBMP15 or mGDF9 alone, but this effect was lost when follicles were also exposed to FSH ( Passos *et al*., 2013 ; Vasconcelos *et al*., 2013 ). It is also important to note that BMP15 and GDF9 from different species appear to activate different second messenger systems, and thus the source of the BMP15 or GDF9 could affect outcomes ( Reader *et al*., 2011 , 2016 ). Granulosa cells collected from small follicles of homozygous Booroola ewes were more responsive to FSH when assessed by stimulation of oestradiol production ( Campbell *et al*., 2006 ). However, it is known that the onset of *CYP19A1* mRNA expression and thus aromatase activity occurs at a smaller follicular diameter in ewes carrying the Booroola mutation than their wild-type contemporaries ( McNatty *et al*., 1985 ; Juengel *et al*., 2017 ). Thus the observed difference in oestradiol response is likely related to increased amounts of aromatase.

## Using knowledge of local regulation of folliculogenesis to improve advanced breeding programmes

Ewes with multiple mutations in genes interacting with the TGFB superfamily can have very high ovulation rates (>10), producing similar numbers of embryos as traditional multiple ovulation embryo transfer (MOET) protocols, providing evidence that modulation of this pathway has the potential to form the basis for a new MOET protocol ( McNatty *et al*., 2017 ). Additionally, these increases in ovulation rate occur without disturbing the normal endocrine patterns of the animal. This is in contrast to what is observed in traditional MOET procedures, which stimulate increased hormonal production from the ovary through elevated FSH concentrations. Therefore reducing the activity of members of the TGFB superfamily potentially will improve oocyte quality and embryo health compared to FSH based protocols ( McNatty *et al*., 2017 ). In both sheep and cattle, immunisation against either BMP15 or GDF9 can increase ovulation rates, inducing a superovulation type effect in some animals ( Juengel *et al*., 2002 , 2004b , 2009 ), without compromising fertilisation or embryo/fetal development ( Juengel *et al*., 2004b ). The challenges with this approach are variable response of the animals to immunisation, both within and between species, resulting in varying efficiencies in neutralisation of bioactivity, coupled with the fact that lack of or very low levels of bioactivity of either GDF9 or BMP15 leads to blockage of ovarian follicular development potentially for months ( Juengel *et al*., 2002 , 2011 ; McNatty *et al*., 2007 ). The extracellular region of the BMPR2 is able to block the proliferative activity of GDF9 & BMP15 combined ( Edwards *et al*., 2008 ). The extracellular region of the BMPR2 fused with an IgG domain can be produced *in vitro* ( Myllymaa *et al*., 2010 ) and might provide a more controlled approach to mimic the neutralising effects of an active immunisation. However, both GDF9 and BMP15 regulate cumulus cell function and have been linked to improved oocyte development when used during *in vitro* oocyte maturation for *in vitro* production of embryos (reviewed in Russell *et al*., 2016 ; Juengel, 2018 ) and thus reducing the bioactivity too far might decrease embryo quality. However, there is no evidence of reduced embryo quality in ewes with reduced GDF9 or BMP15 bioactivity, or mutations in BMPR1B *in vivo* ( Juengel *et al*., 2004b , 2013b , 2018; McNatty *et al*., 2006 , 2017 ), except in heterozygous carriers of the Inverdale mutation undergoing MOET ( McNatty *et al*., 2006 ).

Ewes heterozygous for the Inverdale mutation, or homozygous for the Booroola mutation have increased responsiveness to a MOET protocol than the non-carrier contemporaries ( McNatty *et al*., 2006 ). However, ewes heterozygous for an inactivating mutation in GDF9 did not have increased responsiveness to MOET ( Pinto *et al*., 2018 ). Furthermore, subgroups of animals homozygous for the Booroola mutation have a suppressed responsiveness to FSH-based superovulation procedures potentially through interactions with other, currently unidentified, genetic mutations ( Juengel *et al*., 2013a ). Attempts to mimic the increased responsiveness to FSH-based superovulation protocols in Inverdale ewes using immunisation to decrease BMP15 activity have not been successful ( Juengel *et al*., 2011 ). Additionally, while overall responsiveness to MOET protocols is increased in ewes carrying the Inverdale (I+) or Booroola (BB) mutation, there are still similar levels of variation in response between ewes as observed in wild-type contemporaries. For instance while overall ovulation rate from superovulated homozygous carriers of the Booroola mutation was 26.3 + 1.4, only 59% of the animals produced 4 or more embryos. This is comparable with the 51% of the wild-type contemporaries producing 4 or more embryos ( McNatty *et al*., 2006 ).

## Conclusions and future directions

While it is clear that locally produced factors are critical for regulation of ovarian function throughout the growth and maturation of the ovarian follicle, there is still much to learn. Key mechanisms by which local factors alter ovarian follicular growth and maturation are through regulation of the rate of cell proliferation and responsiveness to gonadotrophins. Here it is clear that members of the TGFB superfamily, particularly BMP15 and GDF9, are key local regulators of follicular development in ruminants, but other family members are also critical and the relative importance of different family members may vary between species, even between closely related ruminants. The potential to use knowledge regarding the local regulators of follicular development to enhance assisted reproductive technologies or treat reproductive pathologies is also present. However, this requires additional understanding of the actions of the local factors and new, cost-effective technologies to modulate these factors *in vivo*.
